# Treatment outcomes in patients with acute thromboembolic occlusion of the superior mesenteric artery

**DOI:** 10.1186/s13019-024-02745-4

**Published:** 2024-04-16

**Authors:** Wenrui Li, Mingyuan Liu, Lei Jin, Hai Feng, Xueming Chen, Zhiwen Zhang

**Affiliations:** Department of Vascular Surgery, Beijing Friendship Hospital, Capital Medical University, Beijing, 100050 China

**Keywords:** Superior mesenteric artery thromboembolism, Acute superior mesenteric artery occlusion, Endovascular treatment, Acute mesenteric ischemia, Opening surgery, Percutaneous mechanical thrombectomy

## Abstract

**Objectives:**

The goals of this study were to investigate the treatment outcomes of acute thromboembolic occlusion of the superior mesenteric artery (ATOS) and identify prognostic factors after treatment.

**Methods:**

The clinical data of 62 patients with ATOS between 2013 and 2021 were retrospectively reviewed. Patients were stratified by the treatment strategy, complications and mortality were compared in different group.

**Results:**

Sixty-two consecutive patients were identified with ATOS. The median patient age was 69 years (interquartile range 58–79 years). Endovascular therapy was initiated in 21 patients, and 4 patients received conservative treatment. Open surgery was performed first in the remaining 37 patients. The technical success rates of the endovascular first group and open surgery group were 90.5% and 97.3%, respectively. One patient in the conservative treatment group had progression of ischemia to extensive bowel necrosis. There was no difference in 30-day mortality between these groups. Predictors of 30-day mortality included initial neutrophil count > 12* 103/dL, age over 60 years old and history of chronic renal insufficiency.

**Conclusions:**

Endovascular treatment or conservative treatment may be adopted in selected patients who do not exhibit signs and symptoms of bowel necrosis, and close monitoring for bowel necrosis is important. The increase in preoperative neutrophil count, age over 60 years old and history of chronic renal insufficiency were poor prognostic factors.

## Background

Acute mesenteric ischemia (AMI) is associated with poor prognosis. It can be caused by mesenteric arterial occlusion, mesenteric venous occlusion, and nonocclusive mesenteric ischemia caused by vasoconstriction secondary to low-flow states [1]. In particular, acute thromboembolic occlusion of the superior mesenteric artery (ATOS) is a life-threatening condition associated with high mortality rates [2]. Early diagnosis and prompt treatment are necessary to prevent bowel ischemia and subsequent bowel infarction, necrosis, or perforation [3]. ATOS is widely recognized as the primary etiology of AMI, whereby superior mesenteric artery embolism (SMAE) is estimated to contribute to approximately 40–50% of AMI cases. The majority of these emboli are believed to originate from a cardiac source, such as atrial fibrillation. Superior mesenteric artery thrombosis (SMAT) may be causative in 25% of cases, and it typically occurs in patients with preexisting atherosclerotic disease within the mesenteric vasculature or accompanied by hypercoagulable states [4–6]. Despite treatment advances and newer techniques, breakthroughs have been made in the treatment of ATOS, and vascular surgeons have both endovascular and open options. The use of endovascular therapy has been increasing in the past decade. However, whether endovascular therapy should be the primary treatment for ATOS is still controversial [6–8]. The goals of this study were to assess the efficacy of different therapies for ATOS.

## Methods

### Patients

This study was an institutional review board-approved study evaluating current treatment for ATOS. A single-institutional procedural database was queried for all consecutive cases of ATOS patients from February 2013 to November 2021. Patients with embolic or thrombotic etiology for AMI confirmed by computed tomography angiography (CTA) or digital subtraction angiography (DSA) examination were included. Patients presenting with AMI secondary to the following conditions were excluded: mesenteric venous thrombosis, nonocclusive mesenteric ischemia, aortic dissections complicated by visceral ischemia, and visceral ischemia occurring as part of an investigational device exemption protocol. The diagnosis of etiology (thrombotic or embolic) was based on the surgeon’s interpretation of the clinical presentation, radiographic findings, and operative findings. We divided the SMA into three numbered segments according to previous study [9].

Previous diagnoses were used to establish conditions such as hypertension, diabetes, hyperlipidemia, smoking history, chronic obstructive pulmonary disease, chronic renal insufficiency, coronary artery disease, congestive heart failure, atrial fibrillation, and cardiac valvular disease. Symptoms on presentation, preoperative imaging, laboratory values, and American Society of Anesthesiologists (ASA) class on admission were recorded.

### Treatment strategies

All patients were seen in consultation with a gastrointestinal surgeon and vascular surgeon. The treatment administered was categorized as endovascular surgery, open surgery and conservative treatment. Initial conservative treatment was indicated selectively in patients with symptoms that were rapidly relieved after admission. The rest of the patients underwent emergency surgery, and the treatment administered was categorized as “endovascular first” or “open surgery”. The endovascular surgery was only performed in patients without evidence of bowel gangrene (e.g., rebound tenderness on physical examination; free air, pneumatosis intestinalis, or mesenteric venous air on CT scan). Open surgery therapy included laparotomy involving surgical embolectomy with or without resection of a nonviable bowel segment.

### Conservative treatment

Conservative treatment encompassed bowel rest, nasogastric drainage, intravenous fluid therapy, parenteral nutritional support, and anticoagulation therapy. If symptoms were aggravated or signs of bowel gangrene were detected, surgical exploration was performed immediately.

### Endovascular treatment

Endovascular therapy included percutaneous mechanical thrombectomy (PMT), catheter-directed thrombolysis (CDT), percutaneous transluminal angioplasty (PTA), and stent implantation. Femoral and brachial access were both used for endovascular therapy. PMT was performed with the 6 F Rotarex System (Straub Medical, Wangs, Switzerland), and small, careful forward and backward passages were slowly performed once or twice. Thrombolysis was performed using a multiple-sidehole infusion catheter (Multi-Sideport, Cook) *via* the SMA with urokinase at a rate of 50,000 IU/h to downsize the emboli. Thrombolysis was monitored by fibrinogen (fibrinogen value was larger than 1 g/L). Heparin was administered uniformly through the arterial sheath. An angiograph of thrombolysis efficiency was performed 48 h after the intervention.

### Open surgery

The decision to perform a laparotomy was made by the operating staff vascular surgeon and colorectal surgeon. The colorectal surgeon also made the decision of bowel viability and the length of the bowel to resect. Embolectomy was performed as the first choice for patients with open surgery, and for patients with failed embolectomy, it was followed by a bypass procedure with an autogenous vein graft. In SMA distal branch embolisms that did not involve the main trunk, embolectomy was not attempted, and only bowel resection was performed.

Technical success of endovascular treatment was defined as the return of bowel perfusion without the need for open revascularization by embolectomy or surgical bypass. The technical success of open surgery was defined as the revascularization of SMA and patient survival at the time of surgery, the revascularization was categorized as embolectomy or bypass grafting. A failed embolectomy followed by a bypass procedure was recorded as a bypass graft. Acute renal failure in the postoperative period was defined as a creatinine > 1.5 mg/dl in patients with normal renal function or an increase of > 20% in patients with chronic renal insufficiency. Pulmonary failure included patients who required intubation > 72 h. Myocardial infarction included electrocardiogram-confirmed ST depression and elevation in the setting of hemodynamic compromise. The diagnosis of stroke was based on clinical examination in conjunction with cerebral imaging. Limited resection was defined by resection with a remaining length of small bowel > 150 cm, which enables ingestion of food without causing short-bowel syndrome. Thirty-day mortality included all deaths within 30 days after treatment.

Patient variables were compared using univariate statistics. Data are expressed as proportions for dichotomous variables and as the mean ± SD or median and interquartile range (IQR) (25– 75th percentiles) for continuous variables. Differences between the groups were determined by the *t* test for parametric data and the Mann–Whitney *U* test for nonparametric data. The χ2 test was used for comparisons of nominal data, and Fisher’s exact test was used when appropriate. Odds ratios (ORs) were used to estimate the differences in the likelihood of death determined by operative, perioperative, and postoperative risk factors. Variables associated with 30-day survival (*P* < 0.1) were entered into a multivariate logistic regression analysis, and significant associations were expressed in terms of odds ratios with 95% confidence intervals (CIs). Statistical significance was set at *p* > 0.05. All analyses were performed using SPSS 24.0 software (SPSS Inc., Chicago, IL, USA).

## Results

Over the 8-year study period, 62 patients with ATOS were treated in our hospital, with 21 (33.9%) patients treated with the endovascular approach first. Open surgery was performed in 37 (59.7%) patients first, and the remaining 4 (6.3%) patients received conservative treatment first. The median patient age was 69 years (IQR, 58–79 years), and 55% were men. The patients’ demographic data, clinical presentations, and characteristics are shown in Table 1. The etiology was embolic occlusion in 45 (73%) patients compared with thrombotic occlusion in 17 (27%) patients. Abdominal pain and emesis were the most common presenting symptoms. The median time between presentation and treatment was 48 h (IQR, 18–120 h). Only minor differences were found between these groups. There was a higher ASA in the open surgery first group (*p* < 0.01) and a trend of higher white blood cell (WBC) count and longer duration of symptoms before treatment, but without a difference in value. According to anatomical classification of SMA, the localisation of occlusions was the proximal SMA (S1) in 13 patients (20.6%), the middle SMA (S2) in 19 patients (30.2%), and the distal SMA (S3) in eight patients (12.7%). It was S1-S2 in six patients (9.5%), S2-S3 in 13 patients (20.6%) S1-S3 in one patient (1.6%), and S1-S2-S3 in two patients (3.2%).

**Table 1:** Demographic and clinical information of patients stratified by treatment type

Variables	All patients(*n* = 62)	Endovascular first(*n* = 21)	Open surgery first(*n* = 37)	Conservative treatment(*n* = 4)	p^a^
Age, median(IQR), y	69(58–79)	65(55–79)	71(61–80)	65(54–74)	0.444
Male, %	55	48	58	75	0.600
Active smoking, %	29	14	38	25	0.193
Comorbidities, %					
Hypertension, %	69	86	59	75	0.095
Diabetes mellitus, %	34	33	38	0	0.269
Atrial fibrillation, %	66	67	65	75	1.000
Coronary artery disease, %	35	48	32	0	0.097
Congestive heart failure, %	13	10	14	25	0.567
Chronic renal insufficiency, %	13	14	11	25	0.459
ASA > 3	27	5	41	25	0.009
Duration of symptoms onset to treatment, median (IQR), h	48(18–120)	24(11–120)	48(24–120)	132(30–168)	0.214
Abdominal pain, %	98	100	97	100	1.000
Emesis, %	61	67	62	25	0.376
Diarrhea, %	35	33	35	50	0.833
Hematochezia, %	23	10	32	0	0.094
WBC count, mean ± SD, × 10^3^/dL	15 ± 9	13 ± 7	17 ± 10	14 ± 8	0.278
Neutrophil ratio median (IQR), %	87(81–90)	87(74–89)	87(82–91)	87(68–92)	0.676
Urea, mean ± SD, mmol/L	9 ± 5	8 ± 6	9 ± 4	11 ± 9	0.679
Creatinine, mean ± SD, umol/L	107 ± 117	126 ± 182	92 ± 35	121 ± 74	0.592
Potassium, mean ± SD, mg/dL	4.0 ± 0.6	3.8 ± 0.6	4.1 ± 0.5	4.2 ± 0.8	0.231
Alanine transaminase, median (IQR), U/L	17(13–30)	15(11.3–28.0)	21.0(12.8–37.3)	20.5(14.0-2649.8)	0.449
Aspartate transaminase, median (IQR), U/L	25(17–48)	22.4(14.4–37.2)	32.4(18.7–54.5)	20.0(16.4-7284.3)	0.294
Lactate, median (IQR), mmol/L	3.3 ± 2.2	2.5 ± 2.5	3.6 ± 2.2	1.5 ± 0.5	0.175
D-dimer (IQR), mg/L	2.3(1.1–4.5)	2.3(1.7–8.5)	2.3(1.1-4.0)	0.9(0.3-8.0)	0.360
Occlusion segmentation of SMA					
S1, %	21	24	16	50	0.224
S2, %	30	42	27	25	0.748
S3, %	13	19	11	0	0.684
S1-S2, %	10	0	16	0	0.138
S2-S3, %	21	15	27	25	0.259
S1-S3, %	2	5	0	0	0.403
S1-S2-S3, %	3	5	3	0	1.000

IQR, interquartile range; SD, standard deviation; ASA, American Society of Anesthesiologists; WBC, white blood cell; SMA, superior mesenteric artery

a Denotes comparisons between these three groups

**Fig. 1:**
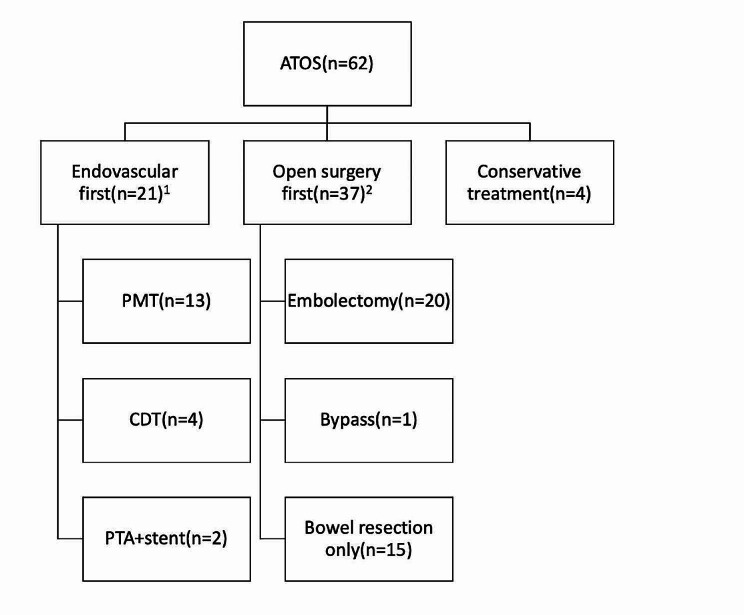
Treatments of patients with ATOS. 1: Two patients had failed endovascular revascularization, 1 patient finally had aorto-SMA bypass with vein and the other had bowel resection alone with extensive bowel necrosis. 2: One patient died with cardiac arrest during the surgery

Descriptive variables for different management are listed in Fig. 1. The technical success rate of the endovascular first group was 90.5%. Of the 2 failed endovascular revascularization patients, 1 patient finally had aorto-SMA bypass with vein and the other had bowel resection alone with extensive bowel necrosis. 19 patients had technical success, 13(61.9%) of them underwent mechanical thrombectomy. 6 of them were treated with mechanical thrombectomy alone, 4 patients were treated with mechanical thrombectomy adjunctive PTA and stenting, and 3 patients had adjunctive CDT after mechanical thrombectomy. For the rest of the patients, four patients had CDT alone. Two patients (9.5%) were treated with primary PTA and stenting. Thirty-seven patients underwent open surgery first, with technical success reaching 97.3%, because 1 patient died with cardiac arrest during the surgery. Bowel resection alone without revascularization was used in 15 patients (40.5%). In these patients, 6 had a limited segment of bowel gangrene, and SMA embolization was restricted to side branches. Nine patients had extensive bowel necrosis, and revascularization was considered to have no effect on improving intestinal blood supply, so they had an extensive bowel resection. A total of 20 (54.1%) patients underwent embolectomy, including 10 who required segmental resection. One patient underwent aorto-SMA bypass with vein grafting and bowel resection. One patient in the conservative treatment first group had progression of ischemia, resulting in open surgery with extensive bowel resection.

The outcomes and post management complications of different management strategies are listed in Table 2. The duration of the initial endovascular procedure was much shorter than that of traditional therapy (95 vs. 200 min; *p* < 0.05), as was the blood loss of these two groups (20 vs. 200 ml; *p* < 0.05). 35% and 25% of the patients avoided bowel resection in the endovascular first group and conservative first group, respectively, which was lower than that in the open surgery first group (70%, *p* = 0.02), and there was no difference in the length of the resected bowel between these groups. Only 1 patient underwent a second-look operation in the open surgery first group because of wound infection, which was much lower than that in the other two groups. However, a trend of higher sepsis was found in the open surgery first group. The endovascular first group and conservative first group had less time needed for bowel rest, stay in the intensive care unit (ICU), and ventilator use. The 30-day mortality rate showed no difference between these 3 groups.

**Table 2:** Outcomes of treatment stratified by treatment type

Variables	Endovascular first(*n* = 21)	Open surgery first(*n* = 37)	Conservative treatment(*n* = 4)	p^a^
Procedure duration, median (IQR), min	95(70–142)	200(170–243)	-	< 0.01
Blood loss, median (IQR), ml	20(15–25)	200(100–300)	-	< 0.01
Ischemic bowel requiring resection, %	35	70	25	0.020
Bowel resection, median, mean ± SD, m	1.43 ± 0.96	2.2 ± 1.2	3.5	0.541
Complications				
Acute kidney injury, %	24	57	25	0.66
Pulmonary failure, %	10	35	0	0.061
Myocardial infarction, %	19	41	25	0.377
Stroke, %	5	5	0	1.000
Second-look operation, %	33	3	25	0.004
Sepsis, %	19	51	25	0.051
Limited resection, %	76	59	75	0.471
Bowel rest time	6(5–10)	11(8–19)	5(3–5)	0.022
Hospital stay median (IQR), day	11(9–17)	18(9–27)	7(5–9)	0.073
ICU time median (IQR), day	0(0–4)	5(1–14)	0(0–3)	0.001
Ventilator used time median (IQR), hour	0(0–5)	11(1–50)	0(0–0)	0.001
Mortality, %	19	43	25	0.200

a Denotes comparisons between these three groups

Further exploration of 30-day mortality risk factors for ATOS patients was performed. An age over 60 years old, history of chronic renal insufficiency, ASA > 3, initial WBC > 10 × 103/dl, neutrophil count > 12* 103/dL, creatinine > 92 µmol/dl, urea > 6.2 mmol/L, D-dimer > 2.6 mg/L and more than one segmentation of SMA involved were associated with an increased risk of death. During the perioperative period, a necrotic bowel also positively affected overall mortality. In the postoperative period, acute renal failure, pulmonary failure, myocardial infarction, and stroke were significantly associated with death. Potential factors before treatment associated with 30-day mortality of these patients were used to create a logistic regression model, and the analysis showed that an initial neutrophil count > 12* 103/dL, age over 60 years old and history of chronic renal insufficiency, were associated with an increased risk of death (Table 3).

**Table 3:** Independent Risk Factors of 30-Day Postoperative Mortality among all patients

Variable	OR (95% CI)	P
Age > 60	30.66(1/18-798.92)	0.040
Chronic renal insufficiency	37.14(1.22-1129.87)	0.038
Neutrophil count > 12* 103/dL	12.88(1.65-100.31)	0.015
D-dimer > 2.6 mg/L	5.60(0.76–41.27)	0.091
More than one segmentation of SMA involved	3.45(0.48–24.79)	0.218

OR, odds radio; CI, confidence interval;

## Discussion

This study evaluated 62 patients of acute thromboembolic occlusion of the SMA over 8 years. Over two-thirds of patients were misdiagnosed with AMI. We report the efficacy of different therapy including endovascular therapy, open surgery and conservative treatment. With a multimodal approach focusing on restoring the blood-flow of nonnecrotic intestine and the removal of nonviable segments of ischemic bowel, as well as medical treatment to prevent progression to multiorgan failure, the 30-day mortality rates were relatively low at 34%. The initial neutrophil count > 12* 103/dL, age over 60 years old and history of chronic renal insufficiency were associated with an increased risk of deathIn past studies, initial conservative treatment was adopted in a selected patient group who had benign clinical symptoms without signs of peritoneal irritation or well-developed collateral circulation [1]. In our cohort, 4 patients had conservative treatment, and all of them had thrombotic occlusion, which may be explained by the fact that the thrombosis was secondary to previous SMA stenosis, so these patients had better collateral circulation than SMAE patients. However, one of the patients had delayed bowel necrosis in 2 days, and extensive bowel necrosis was found in the next surgical laparotomy, which led to multiple organ dysfunction syndrome (MODS) and death. This finding indicates that close monitoring for bowel gangrene is mandatory even in patients with mild symptoms.

For most ATOS patients, more aggressive treatment is necessary, and we have both endovascular and open options. The use of endovascular therapy has been increasing in the past decade, such as percutaneous mechanical thrombectomy, which has been reported for the treatment of ATOS in the form of case reports and case series; these initial studies demonstrate feasibility, but there still remains a large discrepancy in practice patterns [7, 10]. Hypothetically, endovascular treatment has many advantages over open surgery; patients can be treated without the inherent risks of general anesthesia, and it allows immediate and complete assessment of the blood supply before and after intervention [11]. Surgical trauma can be minimized with endovascular techniques and thus reduces infection, the inflammatory response, blood loss and the use of ventilators. These advantages are also shown in our study, with less surgery time, blood loss, bowel rest time, ICU stay time, and ventilator use; however, there was no difference in the 30-day mortality rate between these groups. This is different from some other retrospective studies that had favorable results in terms of mortality or long-term survival rate in the endovascular group [11, 12]. The relatively high mortality rate in the endovascular first group may be because 33% of these patients had delayed bowel necrosis, which led to surgical laparotomy and related death. Nearly half of these patients died because of sepsis and followed MODS. Surprisingly, for the patients who had no bowel necrosis and underwent endovascular treatment, the mortality was much lower (7%), especially compared to the patients in the open surgery first group. The mortality of patients in the open surgery first group was 44% and 40%, respectively, when the patient had or did not have bowel necrosis. In summary, endovascular treatment first could be adopted in selected patients who do not exhibit signs and symptoms of bowel necrosis. In the other hand, an early laparoscopic look would have avoided deterioration of these patients by diagnosing necrotic bowel early in the process, unfortunately, this pattern has not been applied in our center.

For these patients, different kinds of endovascular therapy can be chosen, including PMT, CDT, PTA and stent implantation. Aspiration using a guiding catheter is one of the earliest methods, and other thrombectomy devices have been used in AMI patients, such as the Rotarex debulking Device (Straub Medical, Wangs, Switzerland), AngioJet thrombectomy catheter (Solent Omni; Boston Scientific, Marlborough, MA, USA), and Solitaire FR revascularization device (Covidien, Irvine, CA, USA) [13–16]. The common goal of these treatments is to achieve immediate revascularization of the SMA trunk. We used only Rotarex in this study because we do not have other debulking systems, such as Solitaire FR or Penumbra, and we think Angiojet thrombectomy systems are off label for SMA occlusion. Additionally, a careful operation should be performed when performing PMT to reduce the incidence of SMA pseudoaneurysm or dissection [17]. Therefore, we often use an 8-F introducer if the femoral access is appropriate to use a covered stent in the above situation. Sometimes, CDT is necessary to deal with residual fresh blood clots. On the other hand, the safety of thrombolysis is concerning, mainly regarding hemorrhagic stroke or gastrointestinal bleeding, and it is contraindicated in a patient with recent stroke, recent trauma, cerebrospinal malignancy, or active bleeding [14]. In fact, bleeding complications were relatively low in ATOS patients [8]. In our study, 7 patients had initial CDT or CDT after PMT, and no CDT-related complications were found.

Open surgery should be proactively performed as soon as intestinal necrosis is found. The advantages of open surgery include rapid removal of the necrotic bowel to achieve “damage control” and inspection of the bowel after revascularization. Generally, embolectomy or bypass grafts are two choices to restore blood supply. As in other studies [18, 19], embolectomy was the first choice in our center because embolectomy requires a shorter time for revascularization than bypass surgery. For bypass surgery, the use of prosthetic conduits is contraindicated with intestinal perforation and obvious intestinal contamination. The saphenous vein or femoral vein can be utilized in this setting [14]. In our cohort, 2 cases of aorto-SMA bypass with saphenous vein were performed, including 1 patient who had an endovascular failure.

ATOS patients have a poor prognosis, and several factors may have been considered to affect their postoperative mortality. The first is the etiology; patients presenting with acute thrombosis were found to have decreased survival compared to those with embolus [12, 13]. The result of our study is not consistent with prior studies, with the mortality of thrombosis patients and embolus being 29.4% and 35.6%, respectively (*p* > 0.05). This may be explained by the fact that the thrombosis was secondary to previous SMA stenosis, so some of these patients had basically sufficient collateral circulation, and bowel necrosis can be avoided by even only conservative treatment or endovascular treatment. The treatment outcome of these 9 patients in our study was satisfactory, with no perioperative deaths. On the other hand, the remaining thrombosis patients who suffered from bowel necrosis had poor outcomes, with a 30-day mortality rate of 62.5%. This result is supported by other anatomic autopsy studies where thrombotic occlusions were more likely to be located more proximally than embolic lesions and be associated with prior remote infarcts and aortic wall thrombosis, thus conferring more extensive intestinal infarction [20]. In short, the etiology may affect the extent and severity of ischemic bowel and thus the prognosis. Additionally, it is worth noting that different etiologies can lead to different treatment options; patients with embolic occlusion had a higher rate of treatment by open surgery than patients with thrombotic occlusion (71.1% vs. 41.7%, *P* < 0.01), which is consistent with another study [11].

Second, as we mentioned above, the extent and severity of intestinal ischemia are directly related to patient survival. In AMI patients, leukocytosis or an increase in neutrophil count is often present because partial- or full-thickness bowel necrosis allows bacterial translocation and subsequent leukocytosis ^14^. Similar to another study [21], the increase in WBC count is a predictor of 30-day mortality. In prior studies, other laboratory tests, except leukocytosis, may indicate poor prognosis, such as elevated serum lactate.

The third prognostic factor may be the duration from symptom onset to diagnosis and revascularization. Several early studies reported that patient outcomes significantly improved with diagnosis within 24 h and increased mortality to 70% among patients with a delay of greater than 24 h [12, 22, 23]. However, in relatively recent studies, there was no association between symptom duration and mortality [1, 12]. In this study, there was no improvement in mortality when the patients received treatment within 24–48 h. Obviously, the extent and severity of intestinal ischemia is more important than symptom duration. Apart from that, patients with severe symptoms are more easily diagnosed and receive revascularization faster, but they also suffer from more severe intestinal ischemia, which may lead to bowel necrosis and poor outcomes. In contrast, some patients with rich collateral mesenteric blood flow may present later because of mild symptoms. This finding has been reported previously [11, 24].

Finally, the significant comorbidities present in ATOS patients also contribute to the inability to improve upon outcomes. In our study, the average age of the patients was nearly 70 years old, almost 70% of them had hypertension, and more than 30% had cardiovascular disease. Furthermore, over 10% of patients had significant renal disease at the time of presentation. This is also common in other studies [12]. Old age and comorbidities can make medical treatment fail to prevent progression to multiorgan failure. The logistic regression model proved similar results; age > 60 years old or history of chronic renal insufficiency indicate increased 30-day mortality.

Limitations of this study deserve mention. As a retrospective study, the choice of treatment depended on our judgment at the time. This study is inherently subject to selection bias, as we chose mild and healthier patients with no signs of bowel necrosis to undergo endovascular therapy or conservative treatment while deteriorating patients proceeded to laparotomy directly. Although selection bias was evident, there were few measurable differences between these three groups of patients. Second, we are limited by the relatively small number of patients enrolled, and there may be type II errors. Another limitation is that the long-term survival of patients and quality of life based on treatment were not investigated.

## Conclusion

In conclusion, ATOS continues to be one of the most lethal diseases with high 30-day mortality rates. The outcome of this study showed that an increase in the initial neutrophil count, age over 60 years old and history of chronic renal insufficiency were a poor prognostic factor. Endovascular treatment or conservative treatment may be adopted in selected patients who do not exhibit signs and symptoms of bowel necrosis, and close monitoring for bowel necrosis is important.

## Data Availability

The raw data supporting the conclusions of this article will be made available by the authors, without undue reservation.

## References

[1] Yun WS, Lee KK, Cho J, Kim HK, Huh S (2013). Treatment outcome in patients with acute superior mesenteric artery embolism. Ann Vasc Surg Jul.

[2] Gupta PK, Natarajan B, Gupta H, Fang X, Fitzgibbons RJ (2011). Morbidity and mortality after bowel resection for acute mesenteric ischemia. Surg Oct.

[3] Jia Z, Jiang G, Tian F (2014). Early endovascular treatment of superior mesenteric occlusion secondary to thromboemboli. Eur J Vasc Endovasc Surg Feb.

[4] Clair DG, Beach JM (2016). Mesenteric ischemia. N Engl J Med.

[5] Acosta S (2010). Epidemiology of mesenteric vascular disease: clinical implications. Semin Vasc Surg Mar.

[6] Murphy B, Dejong CHC, Winter DC (2019). Open and Endovascular Management of Acute Mesenteric Ischaemia: a systematic review. World J Surg.

[7] Li W, Cao S, Zhang Z (2022). Outcome comparison of endovascular and open surgery for the treatment of Acute Superior Mesenteric Artery Embolism: a retrospective study. Front Surg.

[8] Zhang Z, Wang D, Li G, Wang X, Wang Y, Jiang T (2017). Endovascular treatment for Acute Thromboembolic occlusion of the Superior Mesenteric Artery and the Outcome comparison between Endovascular and Open Surgical treatments: a retrospective study. Biomed Res Int.

[9] Tual A, Garzelli L, Nuzzo A (2023). Strengthening the Description of Superior Mesenteric Artery Occlusions in Acute Mesenteric Ischaemia: Proposition for an anatomical classification. Eur J Vasc Endovasc Surg Jun.

[10] Zhang Z, Chen X, Zhu R (2017). Percutaneous mechanical thrombectomy treatment of Acute Superior Mesenteric Artery Embolism. EJVES Short Rep.

[11] Block TA, Acosta S, Björck M (2010). Endovascular and open surgery for acute occlusion of the superior mesenteric artery. J Vasc Surg Oct.

[12] Beaulieu RJ, Arnaoutakis KD, Abularrage CJ, Efron DT, Schneider E, Black JH (2014). Comparison of open and endovascular treatment of acute mesenteric ischemia. J Vasc Surg Jan.

[13] Ballehaninna UK, Hingorani A, Ascher E (2012). Acute superior mesenteric artery embolism: reperfusion with AngioJet hydrodynamic suction thrombectomy and pharmacologic thrombolysis with the EKOS catheter. Vascular Jun.

[14] Lim S, Halandras PM, Bechara C, Aulivola B, Crisostomo P (2019). Contemporary Management of Acute Mesenteric Ischemia in the endovascular era. Vasc Endovascular Surg Jan.

[15] Kuhelj D, Kavcic P, Popovic P (2013). Percutaneous mechanical thrombectomy of superior mesenteric artery embolism. Radiol Oncol.

[16] Miura Y, Araki T, Terashima M (2017). Mechanical recanalization for Acute Embolic occlusion at the origin of the Superior Mesenteric Artery. Vasc Endovascular Surg Feb.

[17] Yu Z, Hu J, Lang D (2021). Pseudoaneurysm as a rare complication in the treatment of superior mesenteric artery embolism via percutaneous mechanical thrombectomy: a case report. J Int Med Res Jun.

[18] Batellier J, Kieny R (1990). Superior mesenteric artery embolism: eighty-two cases. Ann Vasc Surg Mar.

[19] Bingol H, Zeybek N, Cingöz F, Yilmaz AT, Tatar H, Sen D (2004). Surgical therapy for acute superior mesenteric artery embolism. Am J Surg Jul.

[20] Acosta S, Ogren M, Sternby NH, Bergqvist D, Björck M (2005). Clinical implications for the management of acute thromboembolic occlusion of the superior mesenteric artery: autopsy findings in 213 patients. Ann Surg Mar.

[21] Chou EL, Wang LJ, McLellan RM (2021). Evolution in the presentation, treatment, and outcomes of patients with Acute Mesenteric Ischemia. Ann Vasc Surg Jul.

[22] Boley SJ, Feinstein FR, Sammartano R, Brandt LJ, Sprayregen S (1981). New concepts in the management of emboli of the superior mesenteric artery. Surg Gynecol Obstet Oct.

[23] Inderbitzi R, Wagner HE, Seiler C, Stirnemann P, Gertsch P (1992). Acute mesenteric ischaemia. Eur J Surg Feb.

[24] Björck M, Acosta S, Lindberg F, Troëng T, Bergqvist D (2002). Revascularization of the superior mesenteric artery after acute thromboembolic occlusion. Br J Surg Jul.

